# Unusual Canal Morphology in Mandibular Premolars With Two Distal and One Mesial Canal: A Case Series

**DOI:** 10.7759/cureus.73832

**Published:** 2024-11-17

**Authors:** Jinesh A, Sanjana Jayakumar Nair, Saurabh Gupta, Harsh Chansoria, Gaurav Rawat

**Affiliations:** 1 Department of Conservative Dentistry and Endodontics, Government College of Dentistry, Indore, IND; 2 Department of Prosthodontics, Government College of Dentistry, Indore, IND

**Keywords:** canal morphology variations, cone beam computed tomography, mandibular premolars, multiple root canals, root canal anatomy

## Abstract

Mandibular premolars exhibit intricate and diverse root canal anatomy, presenting substantial challenges in endodontic treatment. Uncommon variations, such as the presence of three canals, demand meticulous diagnostic and therapeutic approaches to mitigate the risk of overlooking canals and subsequent treatment failure. This case report highlights three cases of mandibular premolars exhibiting a rare root canal configuration with one mesial and two distal canals, successfully managed using cone beam computed tomography (CBCT) for accurate diagnosis and ultrasonic activation for disinfection. The findings underscore the significance of utilizing three-dimensional imaging and precise canal identification in addressing anatomical anomalies in mandibular premolars, which have been sparsely documented in the existing literature. This report aims to contribute to understanding and managing the rare and complex root canal configurations in endodontics.

## Introduction

Mandibular premolars are essential components of the dental arch, playing a key role in the masticatory function and contributing to occlusal stability. The intricate anatomy of root canals has been extensively studied, yet variations continue to challenge traditional endodontic treatment protocols. Numerous studies have investigated the prevalence of root canal morphology variations in mandibular premolars, with particular emphasis on the presence of additional canals [1]. Research findings have revealed a wide range of prevalence rates across different populations, underscoring the influence of ethnic and regional factors on root canal anatomy [2].

The root canal morphology of mandibular premolars can be intricate, which may present a challenge in endodontic diagnosis and treatment. Hoen and Pink [3] found that missed canals (42%) are one of the most common reasons for endodontic failures and re-treatment. Mandibular premolars are often serpentine teeth to treat endodontically [1]. According to Trope et al. [2], it is not uncommon for mandibular premolars to have more than one canal, with racial variation ranging from 15.8% to 39% and an overall occurrence of 28%. A study on the Indian population also discovered that it is not unusual for mandibular premolars to exhibit variation in root canal anatomy, with almost 18% (Vertucci type IV and V) displaying more than one apical opening [4].

This anatomical complexity can result in missed canals and unpredictable treatment outcomes. It is crucial to be mindful of the different root canal configurations to prevent errors. Among the variations encountered, the presence of more than two canals in mandibular premolars represents a significant anatomical anomaly. The prevalence of three root canals varied across different studies. Mengchen Xu et al. [5] reported that up to 0.2% of cases exhibited three canals, with a higher occurrence in first premolars than in second premolars. Meanwhile, Kusai Baroudi et al. [6] found a prevalence of up to 1%. In their research, Elsayed et al. [7] documented a 0.8% incidence of three canals in mandibular premolars, noting the absence of significant variance between genders. Despite its rarity, this configuration demands careful examination and management to ensure successful endodontic outcomes.

Mandibular premolars with three canals and two roots are rare [1]. They may exhibit different configurations, such as two mesial and one distal, one mesiobuccal, one distobuccal and lingual [1], or one mesial and two distal canals [8]. This case report presents three cases of mandibular premolars with one mesial and two distal canals and their management.

## Case presentation

Case 1

A patient sought assistance at the Department of Conservative Dentistry and Endodontics, reporting pain and discomfort in the lower right posterior tooth. The patient had a history of spontaneous, dull pain and food impaction in the same region. There was no significant history of medical illness or systemic disease.

**Figure 1:**
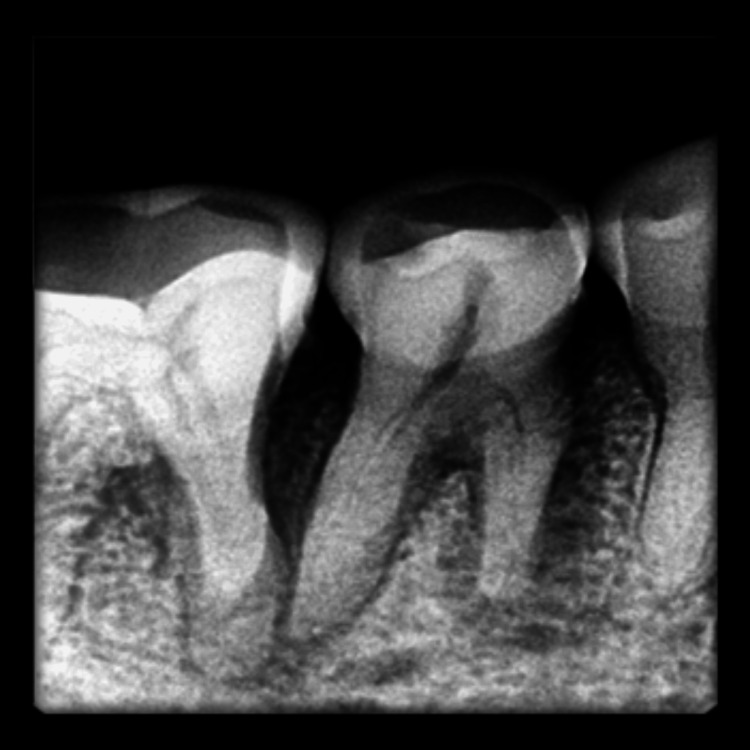
Pre-operative intraoral periapical radiograph of case 1

**Figure 2:**
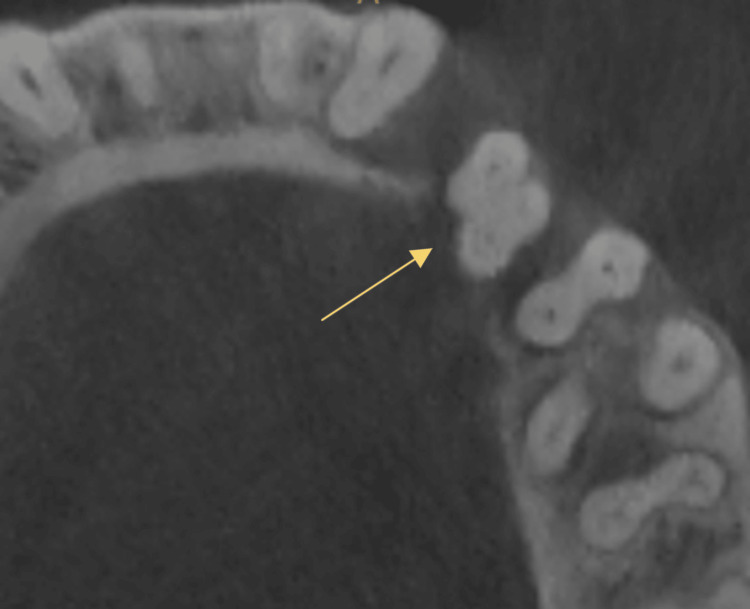
CBCT image showing one mesial and two distal canals of case 1

Clinical examination found no evidence of caries, previous restoration, or fracture. Pain on percussion was present with the mandibular right second premolar. A diagnostic intraoral periapical radiograph revealed two roots and interdental bone loss (Figure 1). The vitality test showed a negative response. Later, a cone beam tomography confirmed the root canal configuration (Figure 2).

The recommended treatment plan was root canal treatment, which was discussed with the patient.

Anesthesia and Isolation

Local infiltration with lignocaine hydrochloride (2% solution, 1.8 mL, epinephrine 1:10,000) was used to anesthetize the tooth.

A rubber dam with a clamp placed on the premolar was used for isolation, followed by an access opening.

Canal Location and Preparation

Following the preparation of the access cavity, the coronal pulp was extirpated. A mesial canal was identified initially, and dentinal mapping revealed an unexpected alteration in trajectory, leading toward the distobuccal and distolingual directions. Coronal flaring was done with the No. 2 Gates Glidden drill (Mani, Inc., Tochigi, Japan). Three canals were located: one on the mesial and two distal. The working length was precisely determined using an electronic apex locator (J. Morita USA, Inc., Irvine, CA, USA) and radiograph. The root canals were negotiated with No. 10 K files (Mani, Inc., Tochigi, Japan), followed by No. 15 and 20.

Irrigation and Obturation

The canals were effectively irrigated using 3% sodium hypochlorite (Prime Dental Products Pvt. Ltd., India) and activated ultrasonically using Ultra X (Eighteenth, Shanghai, China), ensuring thorough cleaning and disinfection throughout the procedure. After the pulp extirpation, the canals were decisively prepared using rotary files (EdgeEndo, Albuquerque, NM, USA). A 17% solution of ethylenediaminetetraacetic acid (Prevest Denpro, India) and saline was also utilized to irrigate the canals, followed by thorough drying using paper points (DiaDent Group International, Cheongju, South Korea). Subsequently, a master cone X-ray was taken, and the canals were obturated using gutta-percha (Dentsply Sirona, York, PA, USA) and sealed with epoxy resin (Meta Biomed, Cheongju, South Korea). The final coronal restoration was done with a nano-hybrid composite (Ivoclar Vivadent, Schaan, Liechtenstein).

**Figure 3:**
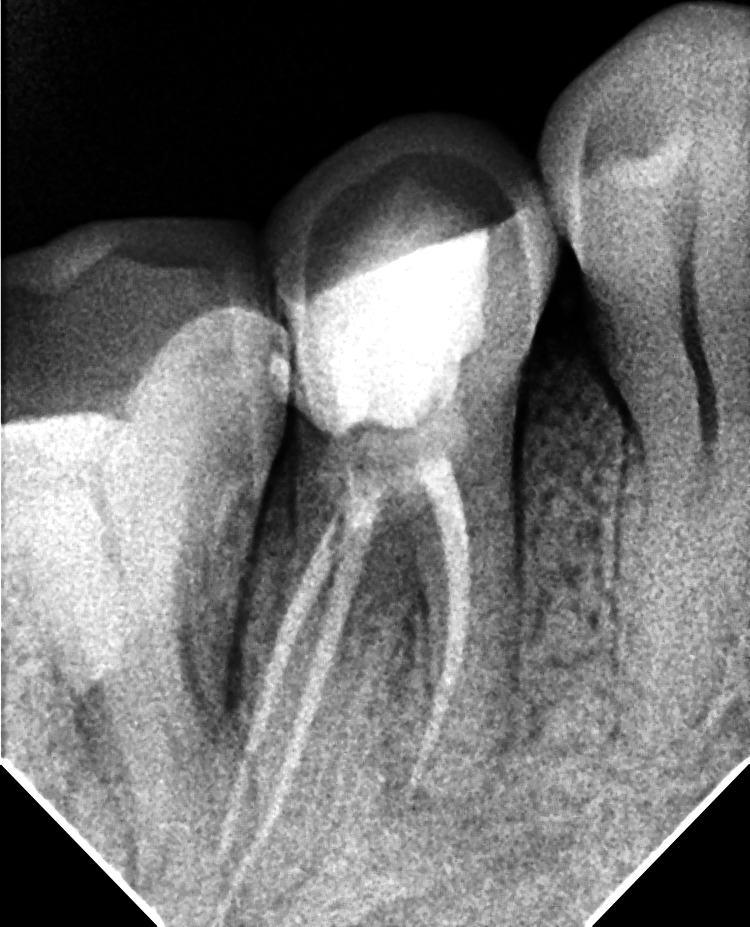
Post-obturation radiograph of mandibular right second premolar of case 1

Case 2

A patient was referred from the Department of Prosthodontics to the Department of Conservative Dentistry and Endodontics. The patient was scheduled for prosthetic rehabilitation, and intentional root canal treatment of the mandibular left and right first premolars was recommended. Following the confirmation of canal anatomy through an intraoral periapical radiograph and cone beam tomography, root canal treatment was scheduled.

**Figure 4:**
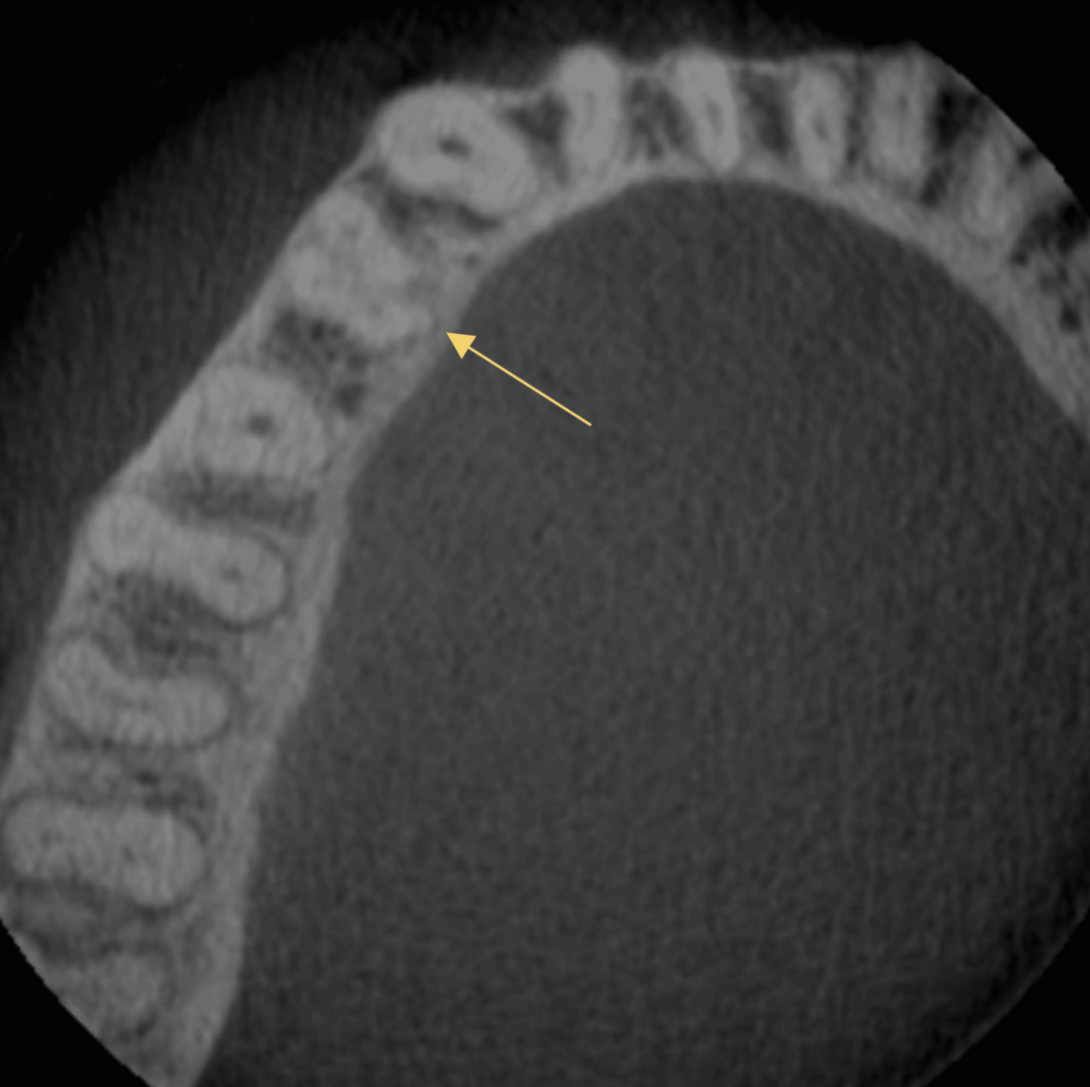
CBCT image of mandibular right first premolar of case 2

**Figure 5:**
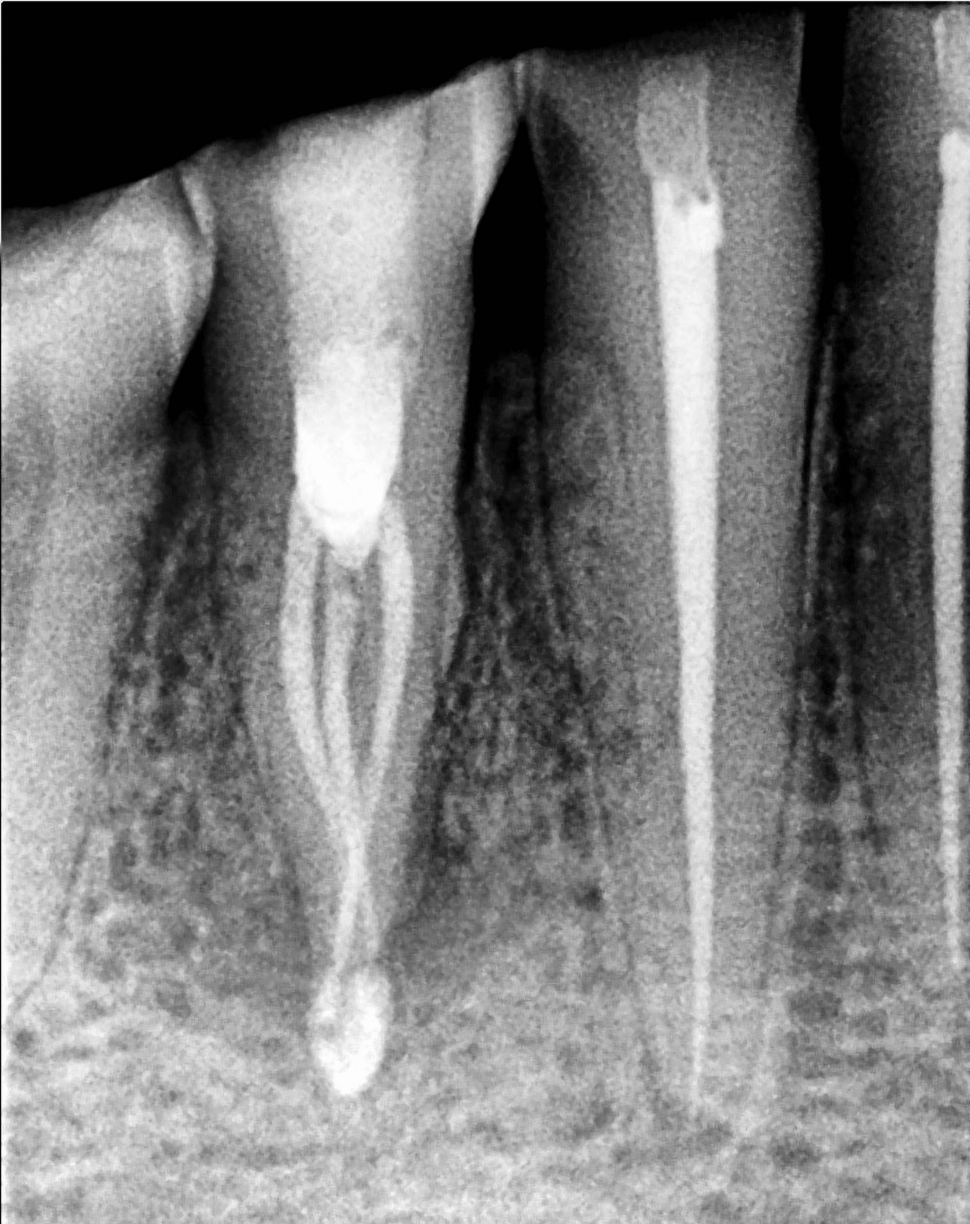
Post-obturation radiograph of the mandibular right first premolar of case 2

**Figure 6:**
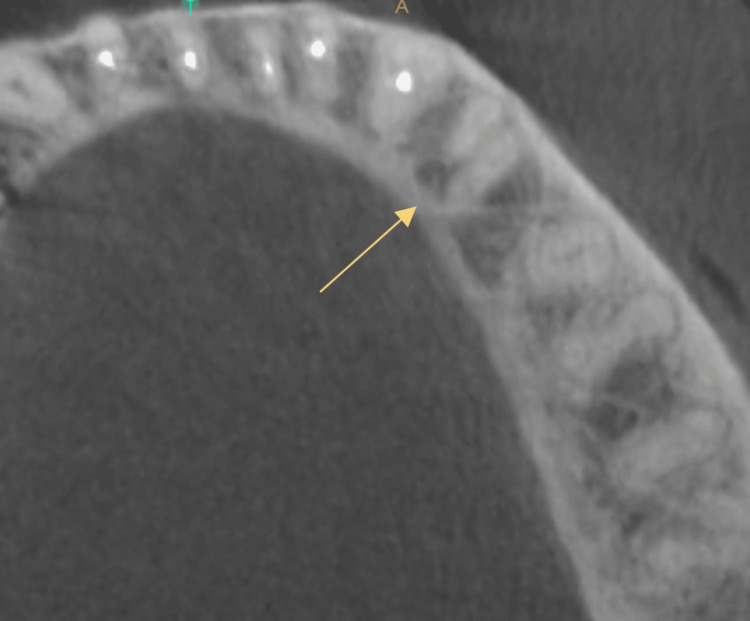
Pre-operative CBCT image of mandibular left first premolar of case 2

**Figure 7:**
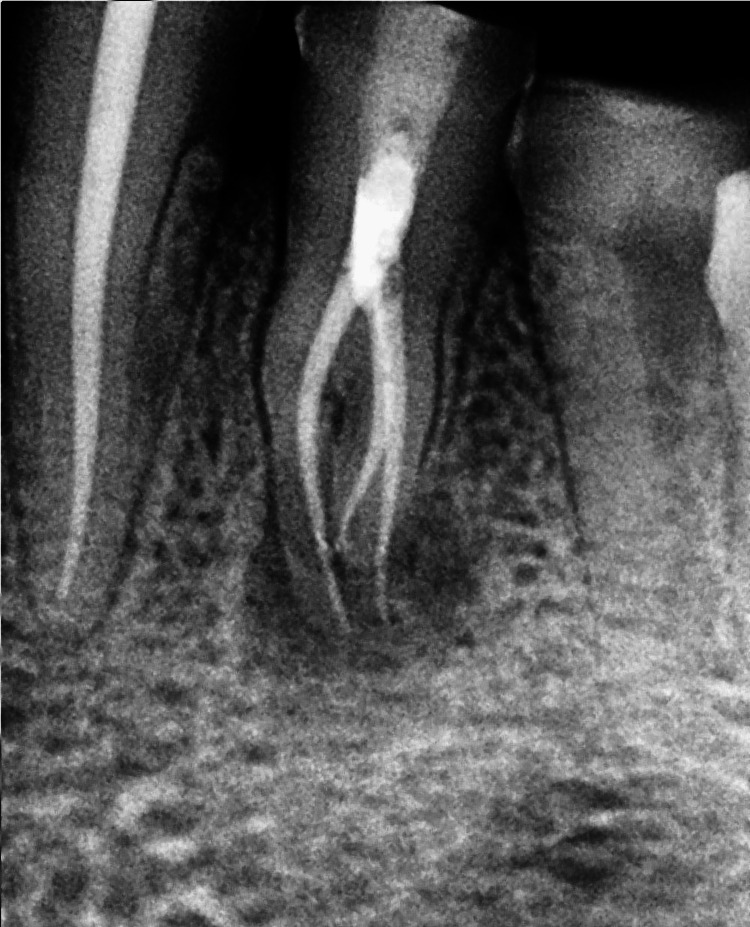
Post-obturation radiograph of mandibular left first premolar case 2

The radiographs confirmed the presence of two roots and three canals in the mandibular right (Figure 4 ) and left first premolar (Figure 6). The root canal procedure was meticulously carried out under rubber dam isolation, and the methods used were consistent with those employed in the previous case. The root canal was later obturated with a bioceramic sealer (Angelus Bio-C Bioceramic Sealer (Angelus, Londrina, Brazil) (Figure 5), (Figure 7).

## Discussion

Mandibular premolars often show aberrant canal anatomy, from single apical or coronal openings to multiple ones [9], making endodontic procedures difficult and unpredictable. Mandibular premolars with three root canals are documented in the literature though they remain a rarity. Rödig and Hülsmann (2003) [10] reported cases of mandibular premolars with three canals, underscoring their uncommon occurrence. Moghadam and Farahi (2021) [11] described such premolars, noting a characteristic widening in the cervical half of the root. While three-canal configurations in mandibular premolars are infrequent, cases presenting with one mesial canal and two distal canals are particularly unusual, as typical configurations more commonly exhibit two mesial canals and a single distal canal. Moayedi and Lata (2004) [12] provided a detailed account of this rare two-mesial and one-distal canal pattern in mandibular premolars, which is consistent with the findings in the present case report. Before the commencement of root-filling procedures, it is imperative to meticulously identify and address any complexities present. Thorough cleaning and disinfection must be diligently carried out to ensure optimal outcomes.

Conventional radiographic examination does not consistently provide an accurate depiction of the internal anatomy of root canals. Consequently, the identification of such variations necessitates the use of three-dimensional tomography, which significantly reduces the risk of overlooking canals. The work of Tachibana et al. (1990) [13] has been pivotal in the advancement of utilizing tomography for the exploration of the internal anatomy of root canals. This pioneering effort has catalyzed substantial growth in the application of microtomography within the field of endodontics [14]. The presence of a sudden interruption in radiolucency within the root canal may indicate the bifurcation or trifurcation of the canal system into two or three distinct canals [15].

Coronal orifice enlargement with Gates Glidden drill was performed before canal negotiation, aiding in anatomical detection and instrument access [16]. This improved visualization helped identify the canal orifices in an anatomical structure with low known prevalence [8]. Sodium hypochlorite was ultrasonically activated to ensure thorough disinfection. The utilization of ultrasonic techniques for the activation of irrigants is integral to the thorough cleansing and disinfection of root canals featuring intricate anatomical structures. This serves to facilitate the dislodgment of debris, disruption of biofilms, and enhancement of the permeation of the solution into anatomically challenging regions [17].

Bioceramic sealer was employed for obturation in two cases due to its biocompatible properties [18], optimal flow characteristics [19], and effective sealing capabilities on atypical root canal surfaces. The presence of less-discussed variations in root canal anatomies in mandibular premolars, as highlighted in our case report, poses a substantial challenge for endodontists. This intricate nature of mandibular premolars renders them a subject of significant interest, and they are aptly described as an enigma for endodontists.

## Conclusions

The complex and variable canal anatomies in mandibular premolars can pose a challenge during endodontic procedures. Utilizing tomography to confirm root canal anatomy and ensuring thorough disinfection can significantly reduce the risk of overlooking canals. Our case report sheds light on the less-discussed mandibular premolars with two distal and one mesial canal, emphasizing the need for further studies in mandibular premolar canal configurations.
